# Short-Fragment DNA Residue from Vaccine Purification Processes Promotes Immune Response to the New Inactivated EV71 Vaccine by Upregulating TLR9 mRNA

**DOI:** 10.1371/journal.pone.0153867

**Published:** 2016-04-15

**Authors:** Jie Shao, Fan Gao, Hui-Juan Lin, Qun-Ying Mao, Pan Chen, Xing Wu, Xin Yao, Wei Kong, Zheng-Lun Liang

**Affiliations:** 1 School of Life Sciences, Jilin University, Changchun, P.R. China; 2 National Institutes for Food and Drug Control, Beijing, P.R. China; University of Massachusetts Medical School, UNITED STATES

## Abstract

To reduce potential oncogenic long genomic DNA in vaccines, nuclease treatment has been applied in the purification processes. However, this action increased the residue of short-fragment DNA and its effect on vaccine potency was still elusive. In this study, we found residual sf-DNA in an inactivated EV71 vaccine could enhance humoral immune response in mice. Ag stimulation *in vitro* and vaccine injection *in vivo* revealed that TLR9 transcription level was elevated, indicating that sf-DNA could activate TLR9. These new findings will help us to understand the molecular mechanism induced by vero-cell culture-derived vaccines.

## Introduction

Toll like receptors (TLRs) have been identified as important links between innate and adaptive immunity, due to their critical roles in mounting protective immune responses against infection and the cross-talk with other pathways with respect to the maturation of dendritic cells (DC) and T cells[1]. Recent studies have revealed TLRs modulated the immune response after vaccination: the yellow fever vaccine (YF-17) activated DC via TLR2,7,8,9 to stimulate proinflammatory cytokines[2]; TLR3 was reported to be involved in the immune response of measles vaccine[3]. In addition, single nucleotide polymorphisms of TLRs have been reported to be able to influence the immune effect of vaccines. For example, TLR2 polymorphisms were associated with non-response to hepatitis B vaccine[4]. Furthermore, TLR agonists have been used as vaccine adjuvants for either prophylactic or therapeutic applications: a TLR4 agonist (3-O-desacyl-4’-monophosphoryl lipid A/MPL) adsorbed to alum known as AS04 is used in a recombinant hepatitis B vaccine (Fendrix^®^, GSK)[5]; CPG 7909, a TLR9 agonist, is applied in an influenza vaccine (Fluarix^®^, GSK)[6]. Dual targeting of TLRs induces synergistic increases in Ag-specific neutralizing antibodies[7].

Vero cells have been used in vaccine production since 1980s[8–15], however, the oncogenic long-fragment residual DNA from vero cell substrates pose a potential risk to human health[16–18]. Thus, nucleases, such as Benzonase manufactured by Merck & Co., were used to digest long-fragment DNA into short-fragment DNA (sf-DNA)[19, 20] which was assumed with better safety[21]. Although drug regulatory authorities have made a specification on the oncogenic long-fragment residual DNA for quality control of vero cell derived vaccines[22, 23], the potential effects of inevitable sf-DNA in final products on immune response remain unknown.

A vero cell-derived EV71 inactivated vaccine, which showed good protection against EV71-associated HFMD in infants and young children in a Phase III clinical trial, was investigated in this study[24, 25]. TLR4 and TLR9 were assumed as potential receptors to recognize the EV71 inactivated vaccine. Because TLR4 was considered to recognize the structural protein of virus[26] which was the main component in the EV71 inactivated vaccine. Short-fragment DNA residue from host cell seemed more likely to activate TLR9 according to a recent study by Hsiao HB, which indicated the released endogenous DNA from dead cells mediated protection against EV71 infection in mice through TLR9 signaling[27]. In this study, we found sf-DNA residue from vero cell modulated immune response to new inactivated EV71 vaccine by upregulating TLR9 mRNA. The results of this study might provide a proof of the positive immune function of residual sf-DNA in immune response to vaccines.

## Materials and Methods

### 1. Inactivated EV71 whole-virus liquid bulk and cell lines

Inactivated EV71 whole-virus liquid bulk (subgenotype C4, vero cell), containing 6000U/ml of Ag, less than 5EU/ml bacterial endotoxin, and with residual vero cell DNA below than 10pg per 400U antigen (Ag) measured by the dot blot hybridization assay following Cao’s[17] protocols, was a gift from Sinovac Biotech. Ltd. (Beijing, China). Vero cells were also obtained from Sinovac Biotech., which were the same as the substrate used for EV71 vaccine production. The cells were maintained in Eagle’s minimum essential medium containing 2% or 10% fetal bovine serum plus 2mM L-glutamine, 100IU of penicillin, and 100μg of streptomycin per ml.

### 2. Mice immunization and serum collection

Normal C57BL/6 mice, TLR4 knockout mice and TLR9 knockout mice on a C57BL/6 background were bred and maintained at the animal facility of National Institutes for Food and Drug Control (NIFDC, Beijing, China). All mice used here were six to eight week-old and specific-pathogen free. The EV71 liquid bulk was diluted to appropriate concentration (25U/ml, 100U/ml and 400U/ml) and then delivered intraperitoneally (i.p.) into female C57BL/6 mice at day 0. PBS was used as negative control and delivered similarly. Blood samples were collected from the female C57BL/6 mice at indicated times (0h, 6h, 12h, 24h, 48h, 72h, 7d and 14d). Blood samples were incubated at 37°C for 1h and then 4°C for 1h before centrifuged at 3000rpm for 10min. The serum samples were inactivated at 56°C for 30min and stored at -20°C until test.

### 3. Ethics statement

All animals were obtained from the Laboratory Animal Center and were maintained in the animal facility of National Institutes for Food and Drug Control (NIFDC, Beijing, China). Protocols for animal use were reviewed and approved by the NIFDC Institutional Animal Care and Use Committee and were conducted in accordance with their guidelines. For the care and use of animals utilized in this study, animals were monitored daily and checked for signs of distress, including hunched posture, respiratory distress, or loss of greater than 15% of initial body mass. All animals were in excellent health and none became severely ill, died or moribund during the whole experiments. All animals were provided with environmental enrichment and food and water were offered *ad libitum*. Animals were considered for euthanasia via an overdose of the anesthetic sodium pentobarbital followed by cervical dislocation, and efforts were made to minimize suffering.

### 4. Neutralizing Antibody

EV71 neutralizing antibody (NTAb) titers were detected using classical cytopathic effect (CPE) assay performed as previously described[28]. Serum samples were inactivated at 56°C for 30min, serially diluted two fold from 1:8 and mixed with equal volumes of TCID50 of a EV71 strain. The mixture was dispensed into a 96-well microplate and incubated at 37°C for 2h. RD cells (1–2*10^5^cells/mL) were added to the mixture. The plates were then placed in a CO_2_ incubator at 35°C for seven days. CPE was observed by microscopy. Chinese national standards of NTAb responses for evaluation of EV71 vaccines were included in each test as a control for the reproducibility of the results[29]. NTAb titers of EV71 were defined as the dilution rate showing 50% inhibition of the CPE. Undetectable antibody values were considered as 4.

### 5. Mice spleen lymphocytes separation and incubation

Stimulation and activation of lymphocytes were performed on individual spleens isolated from C57BL/6 mice. Spleen samples (n = 5 for per group) were homogenized by glass dounce homogenizers in Hank's buffered saline to result in a single cell suspension. Following removal of red blood cells and granulocytes using a Percol density gradient, mononuclear leukocytes were suspended in serum-free medium (Dakewei). Splenocytes were counted before being resuspended to 2*10^6^/ml cells and cultured for 12h at 37°C and 5% CO_2_ before experiment. The spleen lymphocytes were then co-incubated with the EV71 Ag or sf-DNA. Untreated cells were used to determine the background of mRNA transcription. While harvesting, cultures were centrifuged and the precipitations were frozen at -20°C.

### 6. Preparation of sf-DNA

Genomic DNA (gDNA) from vero cell was extracted using Purelink Genomic DNA Kit (Life technologies) following the manufacturer's recommended protocols. Then, 20μg gDNA was incubated with 7.2 units of Benzonase (Merck Millipore Inc.) in a 200μl reaction system at 25°C for 9h. Short-fragment DNA (20~50bp) was separated by polyacrylamide gel electrophoresis and collected by ethanol precipitation. The concentration of gDNA and sf-DNA was quantified by the Qubit 2.0 Fluorometer (Life technologies) with the Qubit dsDNA HS Assay Kit (Life technologies).

### 7. Quantitative real-time PCR

Total RNA extracts were performed using an RNeasy RNA isolation kit (Qiagen Inc.), and the integrity and purity of total RNA were analyzed by agarose gel electrophoresis and NanoDrop UV-spectrophotometer. cDNA was synthesized using the PrimeScript^™^ RT reagent Kit with gDNA Eraser (Takara Inc.). The TLR4 and TLR9 mRNA expression levels were measured by Premix Ex Taq^™^ reaction system (Takara Inc.). The fold change for relative quantification of TLR4 and TLR9 gene expression of post-stimulation relative to untreated control was calculated by the 2^-ΔΔCt^ method using GAPDH as internal reference. Nucleotide sequences of the primers and probes were as follows: GAPDH, F: *5′-CAA TGT GTC CGT CGT GGA TCT-3′*, R: *5′-GTC CTC AGT GTA GCC CAA GAT G-3′*, probe: *5′-(FAM)CGT GCC GCC TGG AGA AAC CTG CC(TAMRA)-3′*; TLR4, F: *5′-ACT CTG ATC ATG GCA CTG TTC TTC 3′*, R: ***5****′-TCC ATG CAT TGG TAG GTA ATA TTA GG-3′*, probe: *5′-(FAM) CCT GCC TGA CAC CAG GAA GCT TGA A (TAMRA)-3′*; TLR9, F: *5′-TGG GCC CAT TGT GAT GAA C-3*, R: *5-TTG GTC TGC ACC TCC AAC AGT-3′*, probe: *5′-(FAM)CCA ACA GTA AGT CTA CGA AGG CTG CCC CAC A (TAMRA)-3′*.

### 8. Statistical analysis

All results were obtained with at least 3 replicates and expressed as the mean ± standard deviation (SD). All statistical analyses were performed using the GraphPad Prism software. Groups were compared using Student’s t-test, and p<0.05 was considered significantly different.

## Results

### 1. Expression of TLR4 and TLR9 mRNA in mice spleen lymphocytes after stimulation with the EV71 inactivated liquid bulk *in vitro* and vaccination *in vivo*

**Fig 1:**
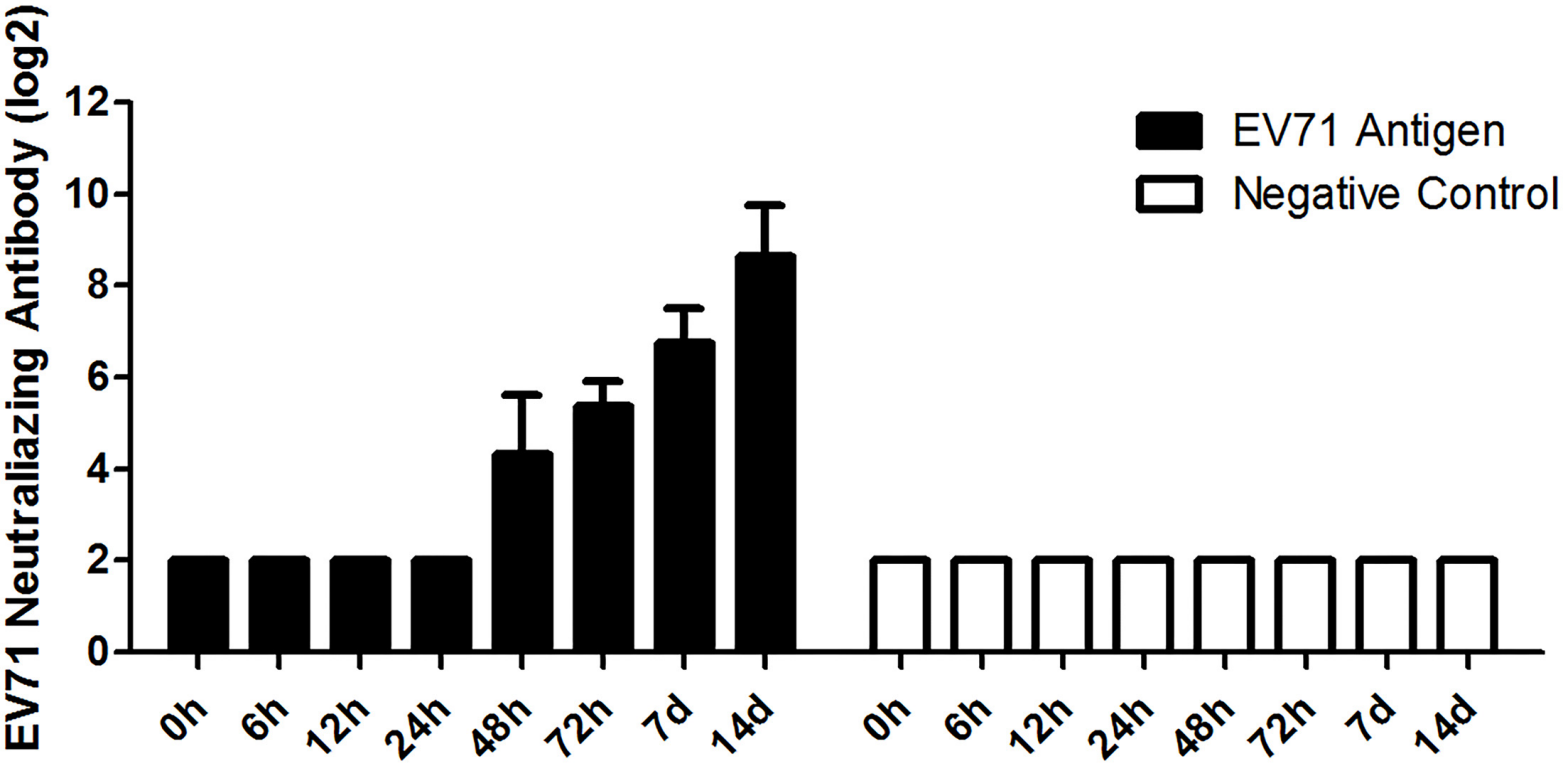
NTAb titer in serum at different time points after vaccination with the EV71 inactivated liquid bulk. Six to eight week-old femal C57BL/6 mice (n = 10 per group) were injected with 400U EV71 inactivated Ag intraperitoneally. PBS was used as negative control and delivered similarly. NTAb titer in serum of each mouse was measured by CPE method at different time points after vaccination (0~14d). The NTAb in mice was initially detected 2d after inoculated, and the NTAb seroconversion rates reached 100% 3d after immunization and persisted up to 14d.

**Fig 2:**
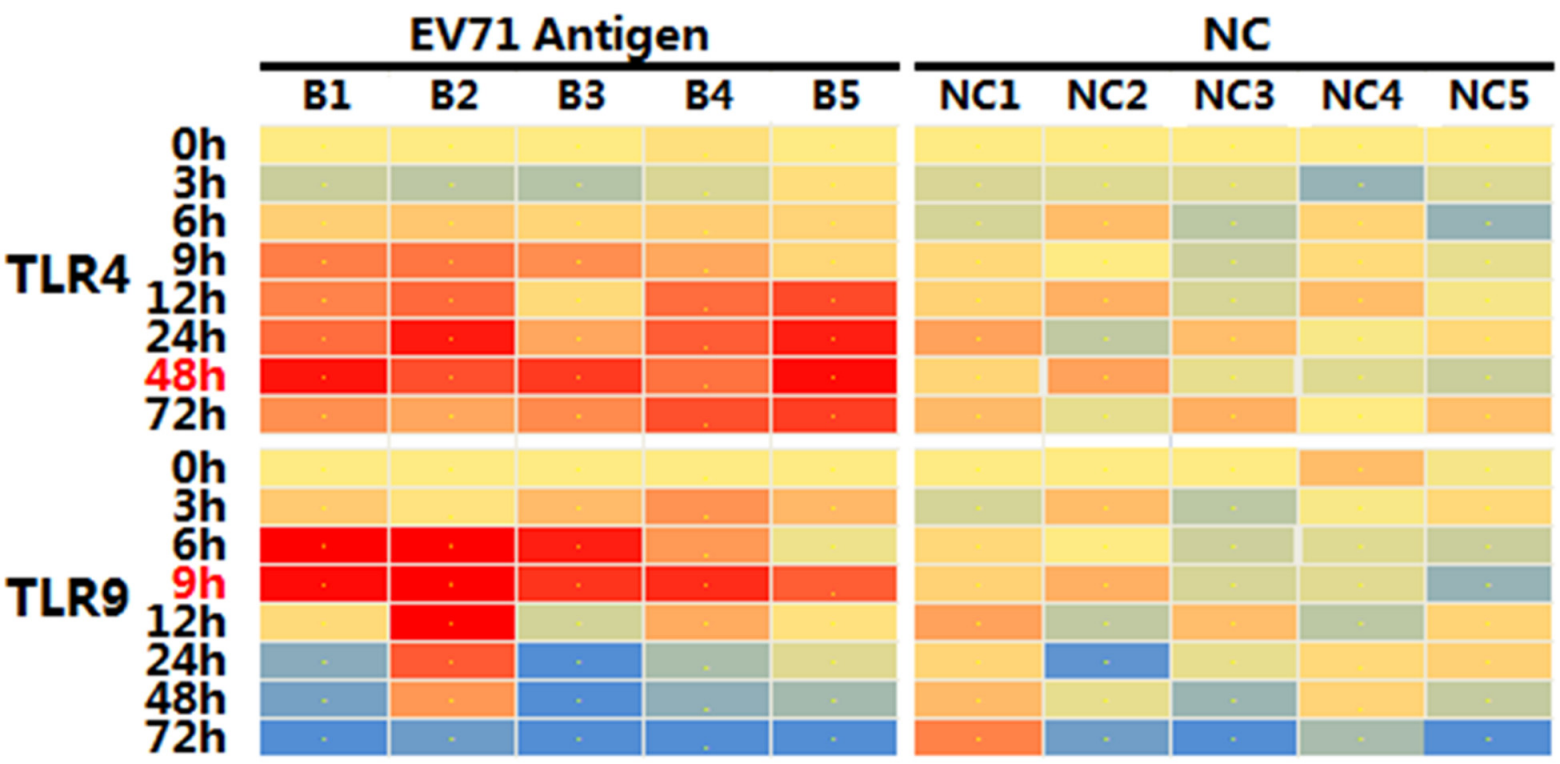
Heat map of TLR4 and TLR9 mRNA expression at different time points after incubation with EV71 inactivated liquid bulk *in vitro*. Each column in the graph represents a mouse (n = 5 per group), and colors ranging from blue to red corresponds to the fold change of the TLR4 or TLR9 mRNA expression after incubation with 100U/ml EV71 inactivated Ag. Medium was used as negative control and incubated with spleen lymphocytes similarly. The TLR4 mRNA expression peaked at 48h after incubation, while TLR9 peaked at 9h for most samples.

**Fig 3:**
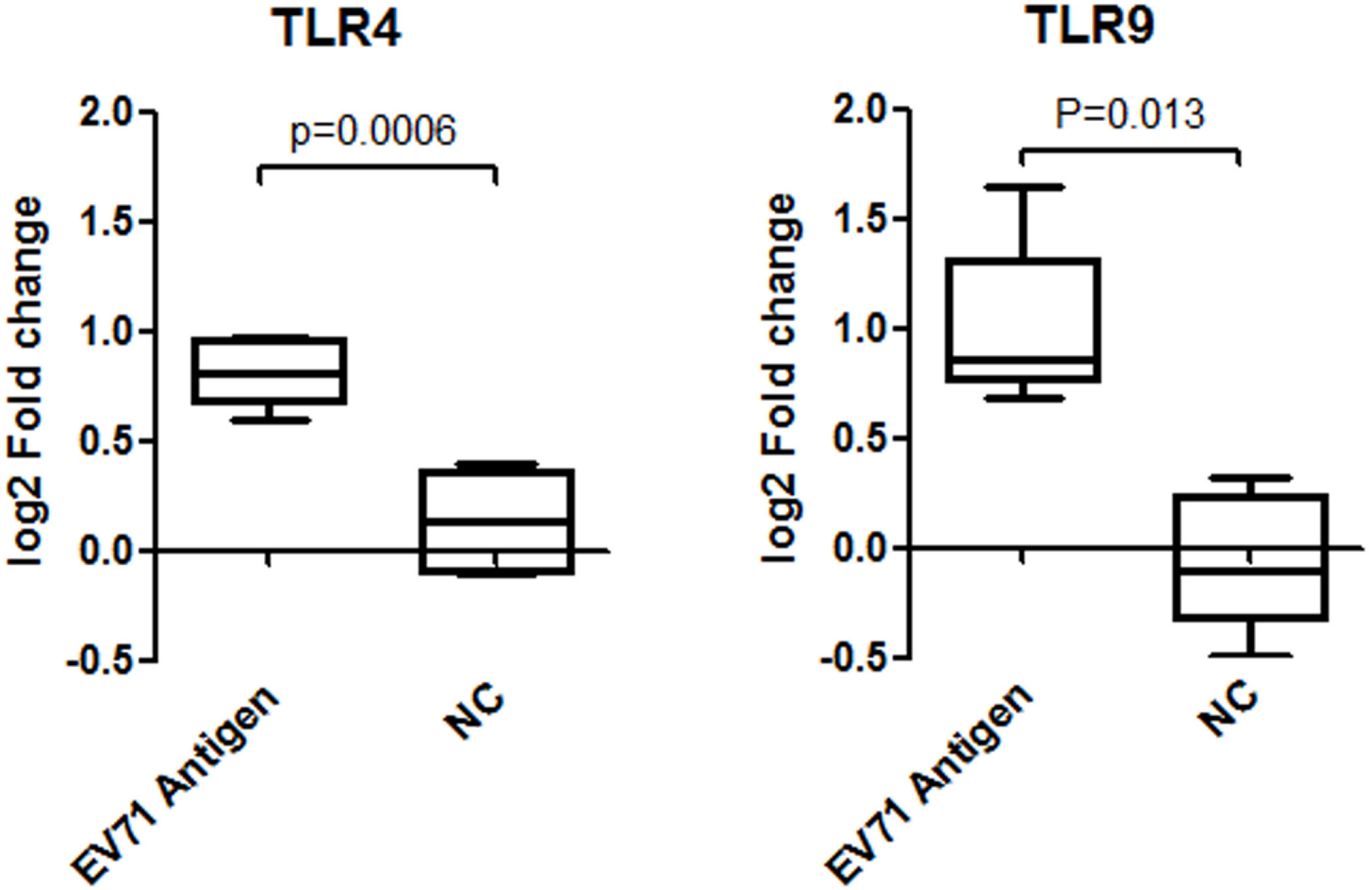
TLR4 and TLR9 mRNA expression after stimulated with EV71 inactivated liquid bulk *in vitro*. TLR4 mRNA expression was tested by q-PCR at 48h after incubation with 100U/ml EV71 inactivated liquid bulk, while TLR9 was measured at 9h. Significantly increased expression of TLR4 mRNA (n = 5, fold increase = 1.76 *p* = 0.0061) and TLR9 mRNA (n = 5, fold increase = 1.91, *p* = 0.0013) was observed in mice spleen lymphocytes after stimulation with EV71 inactivated liquid bulk.

Since the NTAb in mice could be detected as early as 2d after vaccination, and seroconversion rates reached 100% at 3d after immunization (Fig 1), we analyzed the expression levels of TLR4 and TLR9 mRNA using a q-PCR approach at 8 different time points within 3 days to explore the dynamic changes of TLR4 and TLR9 in response to EV71 Ag. Results showed that the expression of TLR4 mRNA increased after incubation with the EV71 inactivated Ag, and reached the peak value for most samples at 48h after stimulation (Fig 2). But it peaked at 9h for TLR9 (Fig 2). A further analysis revealed significantly higher levels of TLR4 and TLR9 mRNA compared to negative controls (Fig 3; TLR4, n = 5, fold increase = 1.76, *p* = 0.0061; TLR9, n = 5, fold increase = 1.91, *p* = 0.0013).

**Fig 4:**
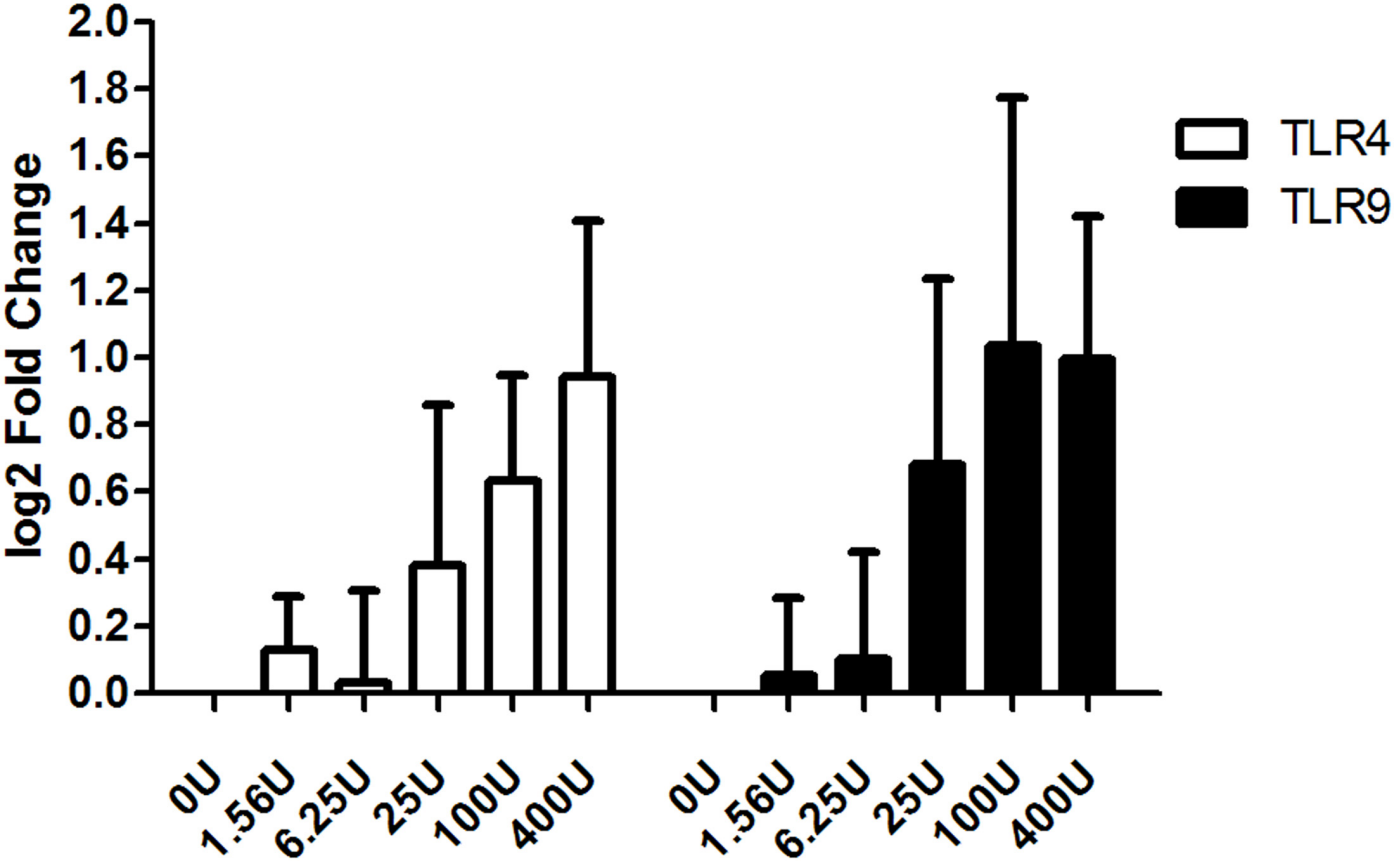
Dose-response effect between the expression of TLR4,9 and the concentration of the EV71 inactivated Ag. Five serial dilution concentrations of EV71 liquid bulk (400~1.56U/ml) were used to incubate with mouse spleen lymphocytes *in vitro* (48h to TLR4 test and 9h to TLR9 test) (n = 5 per group). Clear dose-response relationships were observed between TLR4,9 mRNA and EV71 Ag concentrations.

Five different concentrations of EV71 Ag (1.56~400U) were used to explore the dose-response effect between the expression levels of TLR4,9 and concentrations of EV71 Ag. As shown in Fig 4, there were clear dose-response relationships between TLR4,9 and EV71 Ag.

**Fig 5:**
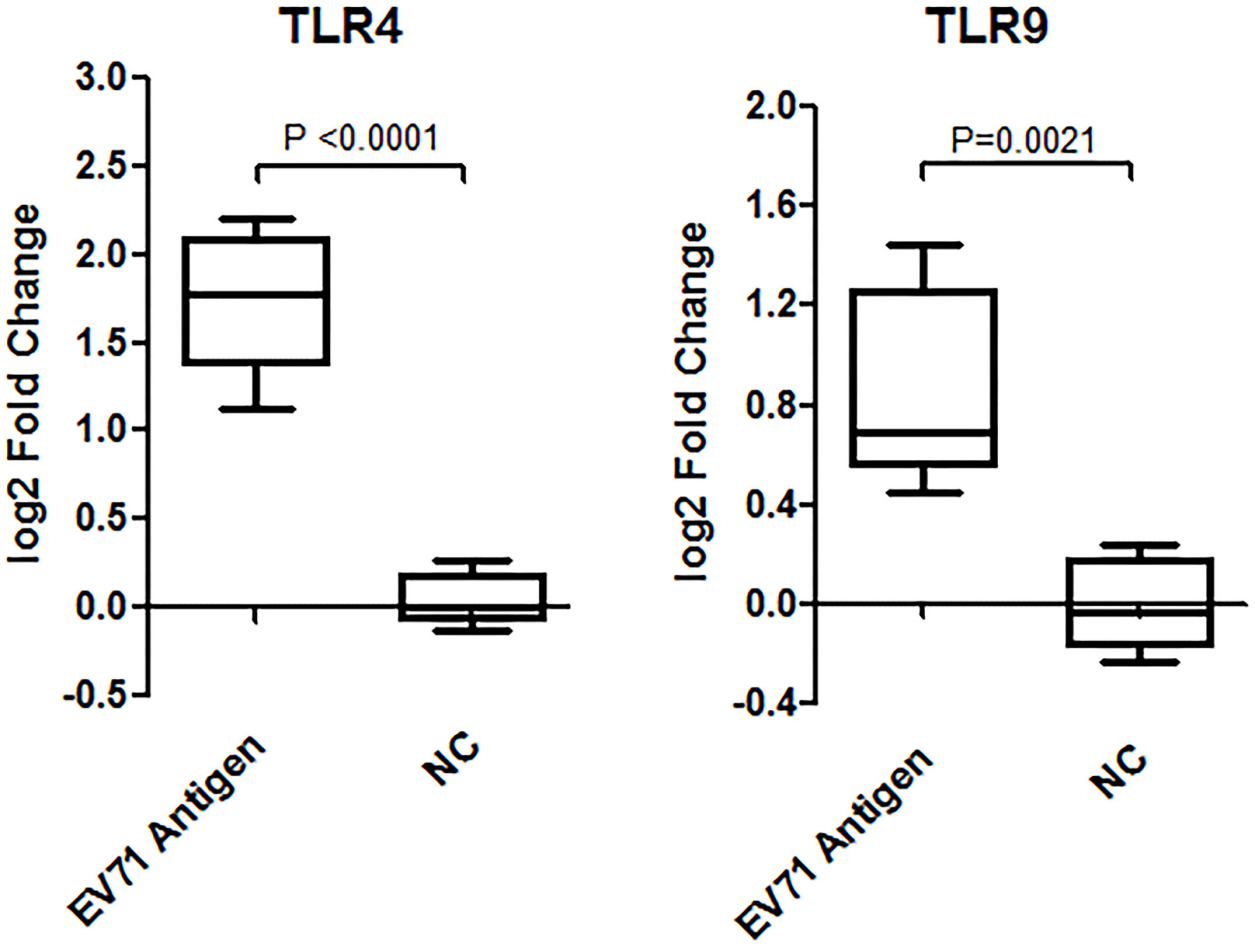
TLR4 and TLR9 mRNA expression after vaccination with the EV71 inactivated liquid bulk *in vivo*. TLR4 mRNA expression was tested by q-PCR at 48h after vaccination with 400U Ag for each mouse, while TLR9 was measured at 9h. Significantly increased expression of TLR4 mRNA (n = 5, fold increase = 1.87, *p*<0.0001) and TLR9 mRNA (n = 5, fold increase = 3.06, *p* = 0.0021) was observed in mice spleen lymphocytes after vaccination with the EV71 inactivated liquid bulk.

To verify our findings *in vitro*, the mRNA expression levels of TLR4 and TLR9 in mice spleen lymphocytes after vaccination with EV71 Ag *in vivo* were explored. Results showed a significantly up-regulated of both TLR4 and TLR9 mRNA after vaccination with EV71 liquid bulk compared to samples vaccinated with PBS (Fig 5; TLR4, n = 5, fold increase = 1.87, *p*<0.0001; TLR9, n = 5, fold increase = 3.06, *p* = 0.0021).

### 2. Cytokines activated by TLR4 and TLR9 signaling were up-regulated after incubation with EV71 inactivated liquid bulk

**Fig 6:**
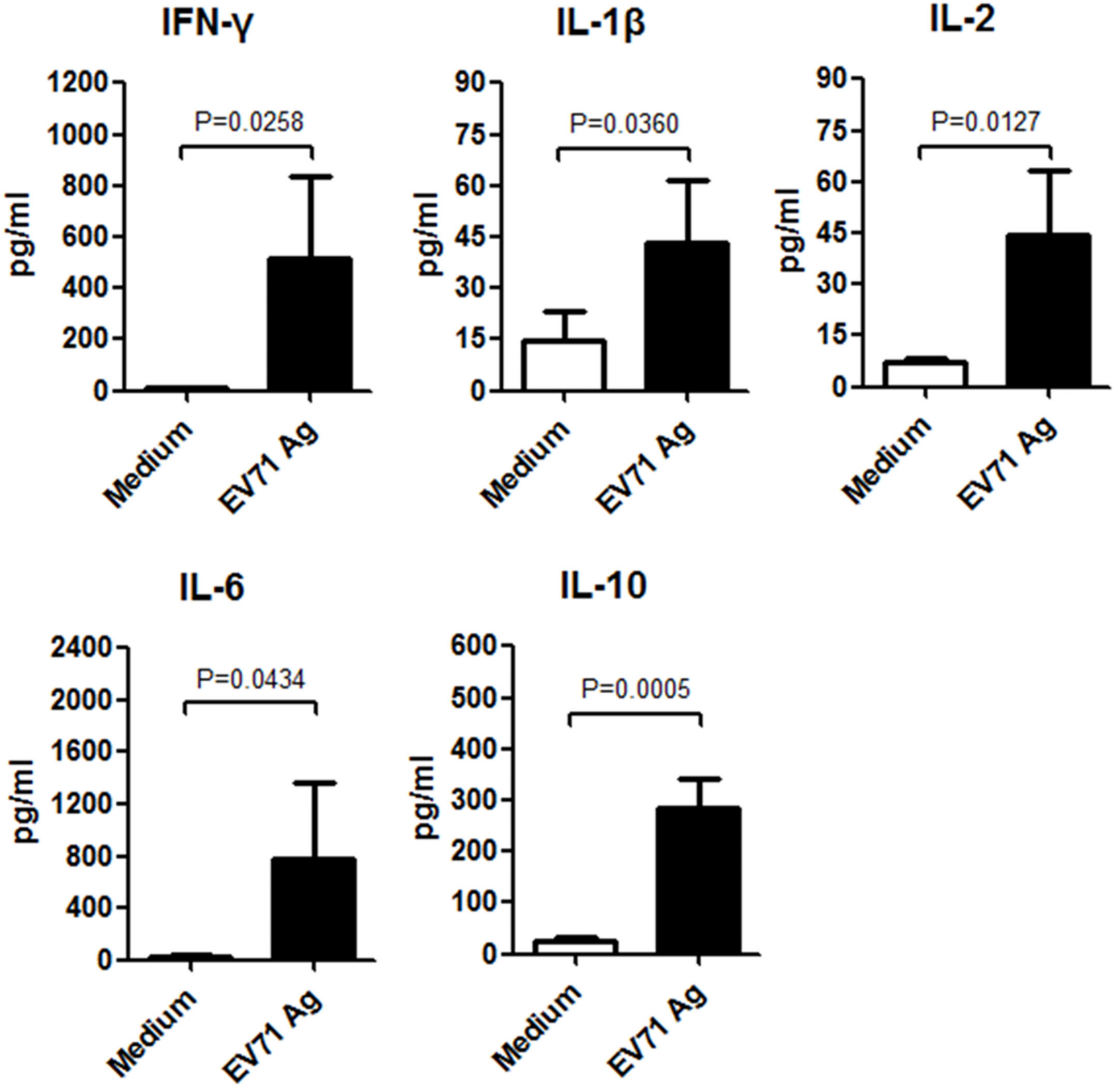
Secretion of cytokines in culture supernatants of spleen lymphocytes after stimulation with EV71 inactivated liquid bulk. Spleen lymphocytes from C57BL/6 mice (n = 5 per group) were co-incubated with EV71 inactivated liquid bulk (100U/ml) for 48h. Untreated samples were used to determine the background of secretion of cytokines. Suspension was then centrifuged, and cytokines in the cell-free supernatants were tested by luminex method. Five cytokines (IL-1β, IL2, IL6, IL-10 and IFN-γ) were found to be significantly up-regulated after incubation.

Activation of TLR4,9 signaling mediated the production of cytokines necessary for the development of effective immunity. So the secretion level of 23 cytokines in culture supernatants of spleen lymphocytes after incubation with EV71 Ag for 48h were tested by the luminex method (Bio-Plex Pro^™^ Mouse Cytokine 23-plex Assay). The secretion of five cytokines (IL-1β, IL2, IL6, IL-10 and IFN-γ) were found to be significantly increased (Fig 6). All the cytokines mentioned above have been reported could be activate by TLR4 and TLR9 signaling[7].

### 3. NTAb responses to the EV71 inactivated liquid bulk in TLR4 gene knockout and TLR9 gene knockout mice

**Fig 7:**
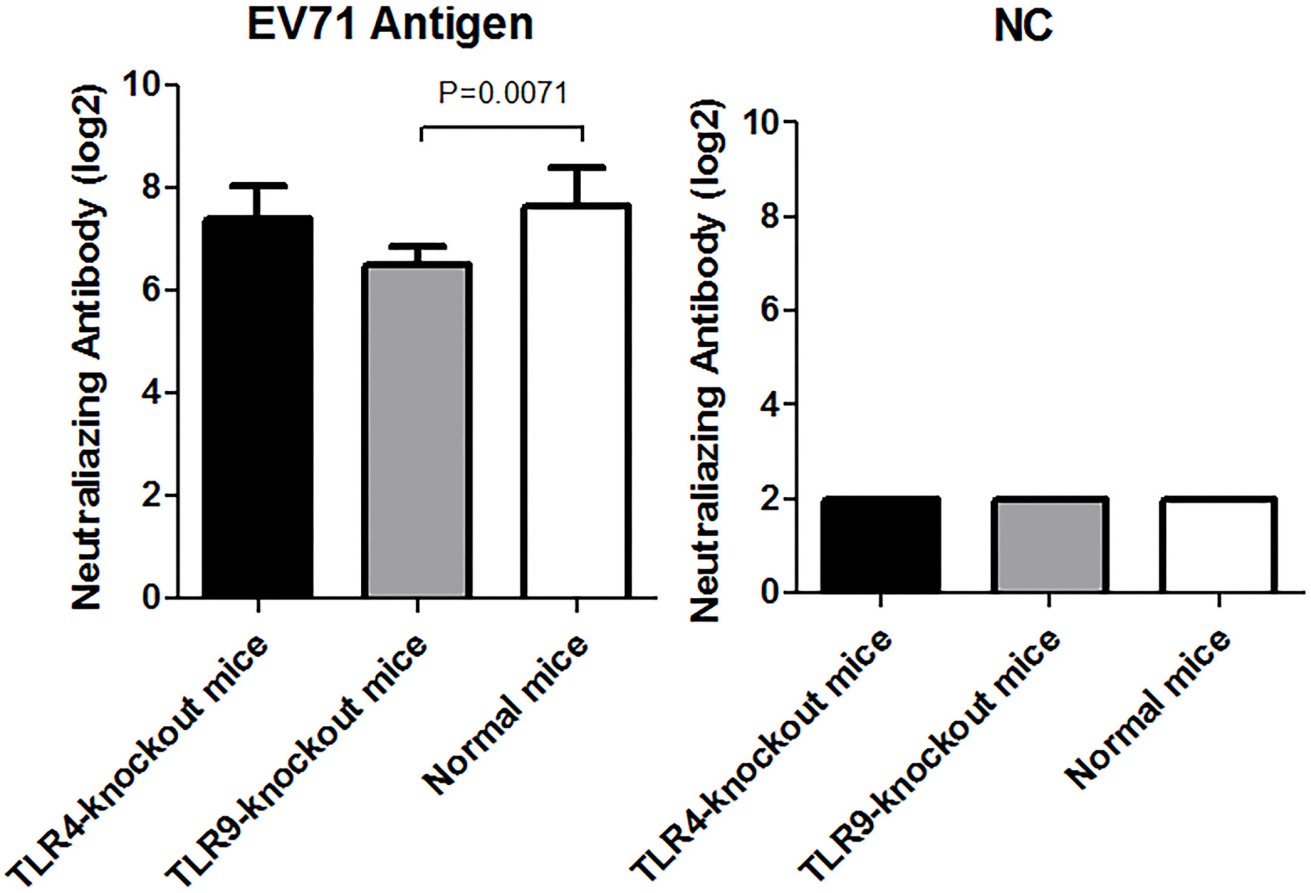
NTAb titer in normal, TLR4 gene knockout and TLR9 gene knockout mice after vaccination with EV71 inactivated liquid bulk. Each mouse was injected with 400U EV71 inactivated liquid bulk (n = 10 per group), and NTAb level was tested 7d after vaccination. Immunization and NTAb test methods were similar to Fig 1. A significant difference in NTAb titer was found between TLR9 gene knockout mice and normal mice (n = 10, *p* = 0.0071).

The results above suggested that TLR4 and 9 might be involved in the immune response to EV71 inactivated liquid bulk. To investigate the relationships between TLR4,9 and the immune effect of the EV71 Ag, NTAb levels were measured in the TLR4 gene knockout and TLR9 gene knockout mice at 7d after vaccination with 400U EV71 inactivated liquid bulk, and normal mice were treated as control. Lower NTAb levels were observed in both TLR4 gene knockout and TLR9 gene knockout mice compared to normal mice (Fig 7). However, significant difference was only found between TLR9 gene knockout mice and normal mice (n = 10, *p* = 0.0071). This findings well supported the above results, which was a strong evidence that TLR9 played an important role in response to the EV71 inactivated vaccine.

### 4. TLR9 mRNA expression in mice spleen lymphocytes after stimulation with vero cell sf-DNA *in vitro*

**Fig 8:**
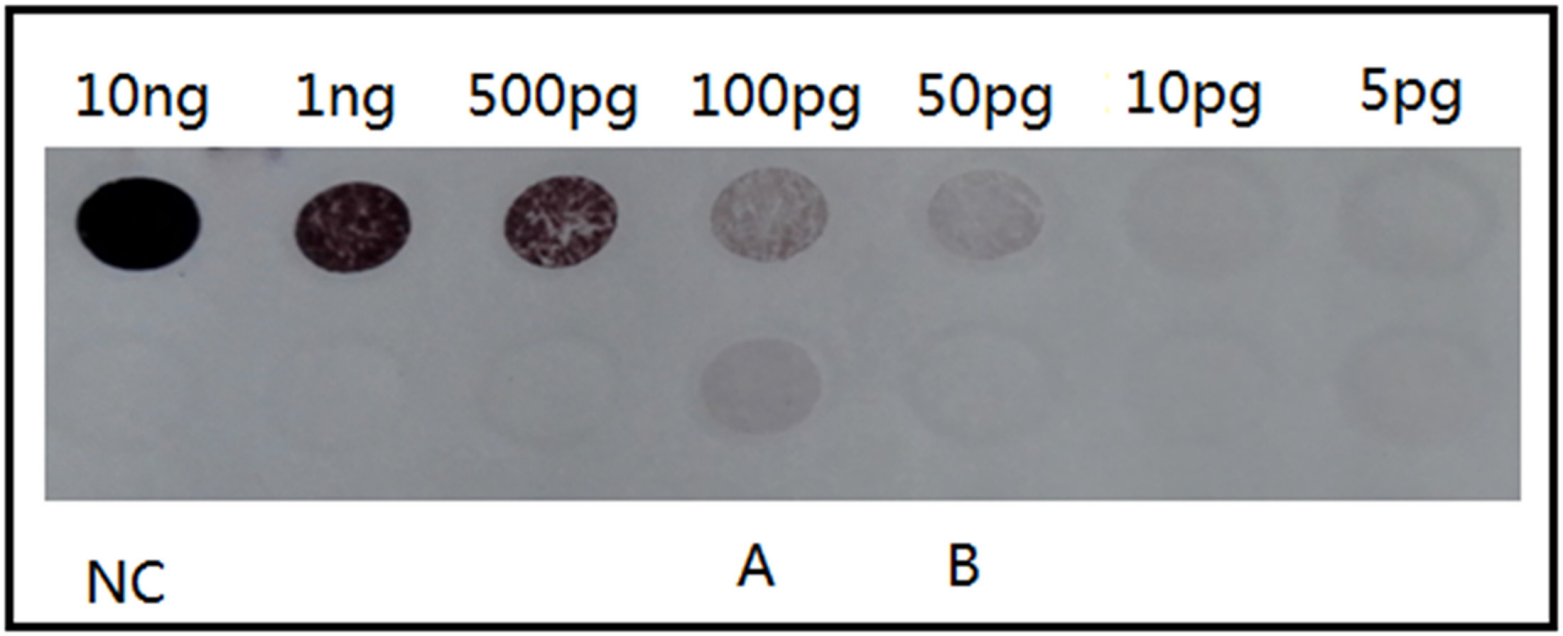
Dot blot hybridization assay of residual vero cell DNA in the EV71 inactivated liquid bulk and the purified sf-DNA solution. Dot blot hybridization assay was used to measure vero cell DNA in the EV71 inactivated liquid bulk and the purified sf-DNA solution, and vero cell DNA reference standards facilitated the standardization. The result showed less than 10pg DNA in 400U EV71 inactivated liquid bulk (marked B in the figure), and about 50pg DNA in each microliter purified sf-DNA solution (marked A in the figure).

TLR9 was considered to recognize unmethylated CpG sequences in DNA molecules. Since EV71 belongs to the *Picornaviridae* family and has RNA as its genetic material, gDNA from host cell was the only source of DNA components in EV71 vaccine[20]. However, long-fragment DNA (>50bp) was shown less than 10pg in per 400U EV71 inactivated liquid bulk tested by dot blot hybridization assay (marked B in Fig 8) which was recommended by the Pharmacopoeia domestic and overseas. Thus, the residual sf-DNA (<50bp) was assumed as the potential component that triggered the activation of TLR9 in the EV71 inactivated bulk. The concentration of sf-DNA was measured by the Qubit dsDNA HS assay due to this method can detect shorter DNA in theory and there was no other suitable method available. The results showed 1510 pg in per 400 U EV71 Ag.

**Fig 9:**
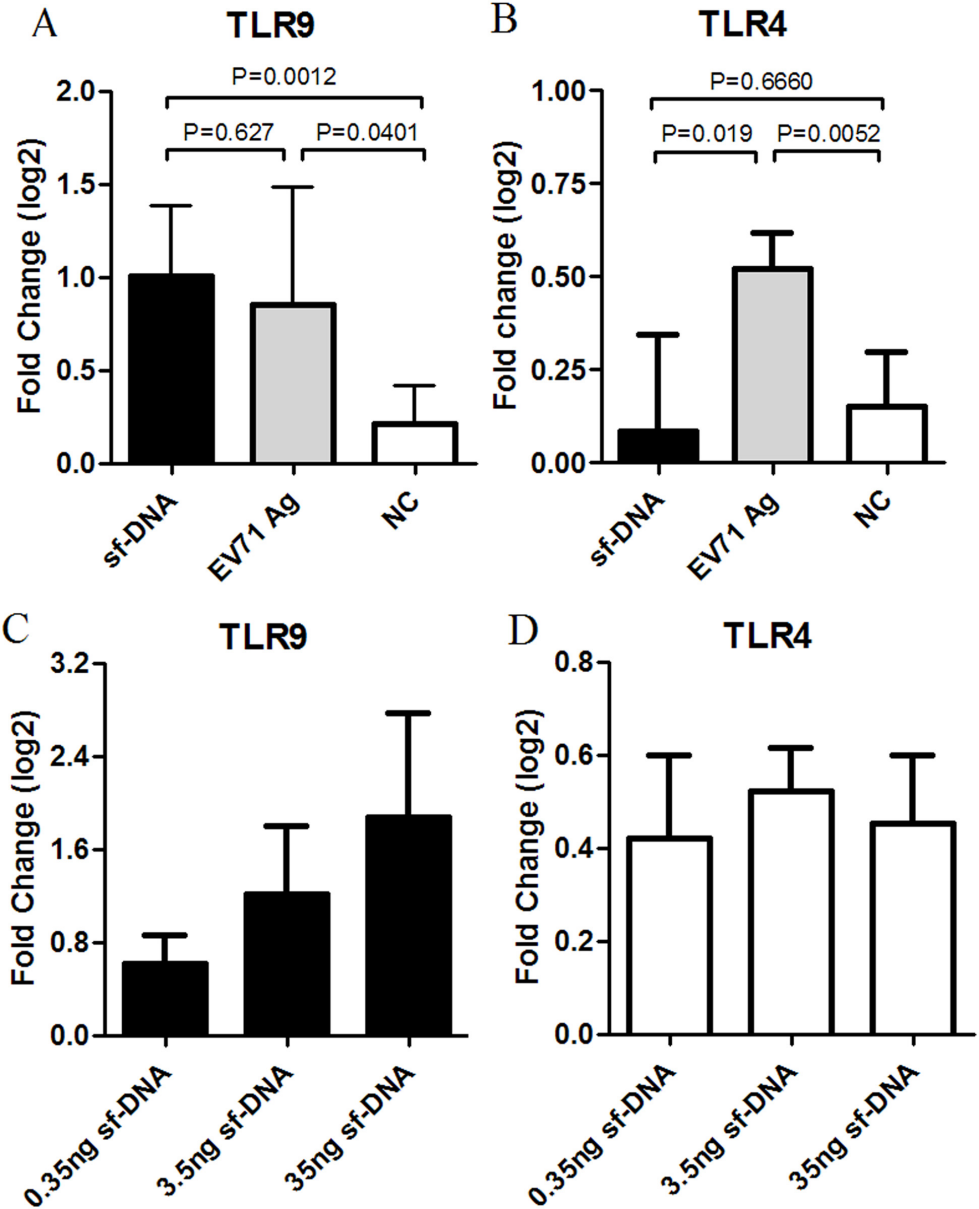
Expression level of TLR4 and TLR9 mRNA in mice spleen lymphocytes after stimulation with sf-DNA. The sf-DNA incubation and mRNA expression test methods were similar to Fig 3. Significantly increased expression of TLR9 mRNA was found both after incubation with 0.38ng/ml sf-DNA (n = 6, *p* = 0.041) and 100U/ml EV71 inactivated Ag (n = 6, *p* = 0.0012), but there was no significant difference between sf-DNA and EV71 inactivated Ag group (n = 6, *p* = 0.627) (Fig 9A). Short-fragment DNA didn’t increase the expression of TLR4 (n = 6, *p* = 0.6660) (Fig 9B). Three serial diluted concentrations of sf-DNA (0.38~38ng/ml) were added into 100U EV71 Ag to incubate with mice spleen lymphocytes (48h to TLR4 test and 9h to TLR9 test). A clear dose-response relationship was found between TLR9 mRNA and sf-DNA concentration (n = 6 per group) (Fig 9C). But there was no dose-response effect between TLR4 and sf-DNA concentration (n = 6 per group) (Fig 9D).

Purified sf-DNA (20~50bp) was prepared to verify our hypothesis, and the sf-DNA concentration was about 50pg/μl tested by the dot blot hybridization assay (marked A in Fig 8) and 2500pg/μl by the Qubit dsDNA HS assay. The huge gap between these two results showed Qubit dsDNA HS assay was capable of detecting shorter DNA fragments. The expression levels of TLR9 mRNA in mice spleen lymphocytes were measured after stimulation with this purified sf-DNA. Results showed a significantly increased expression of TLR9 mRNA (Fig 9A; n = 6, *p* = 0.0401) after incubation with sf-DNA at a final concentrations of 380pg/ml that was equal to the content of sf-DNA in 100U EV71 inactivated Ag. However, there was no significant difference between the sf-DNA and EV71 Ag treated groups (Fig 9A; n = 6, p = 0.6270). This result accorded with our former hypothesis.

Three different concentrations of sf-DNA (0.38ng/ml, 3.8ng/ml and 38ng/ml) were added to 100U EV71 Ag to explore the dose-response effect between the expression of TLR9 and the concentration of sf-DNA. And a clear dose-response relationship was observed (Fig 9C). But there was no dose-response effect between TLR4 and sf-DNA concentration (Fig 9D).

### 5. NTAb responses to the EV71 inactivated liquid bulk adjuvanted with sf-DNA in mice

**Fig 10:**
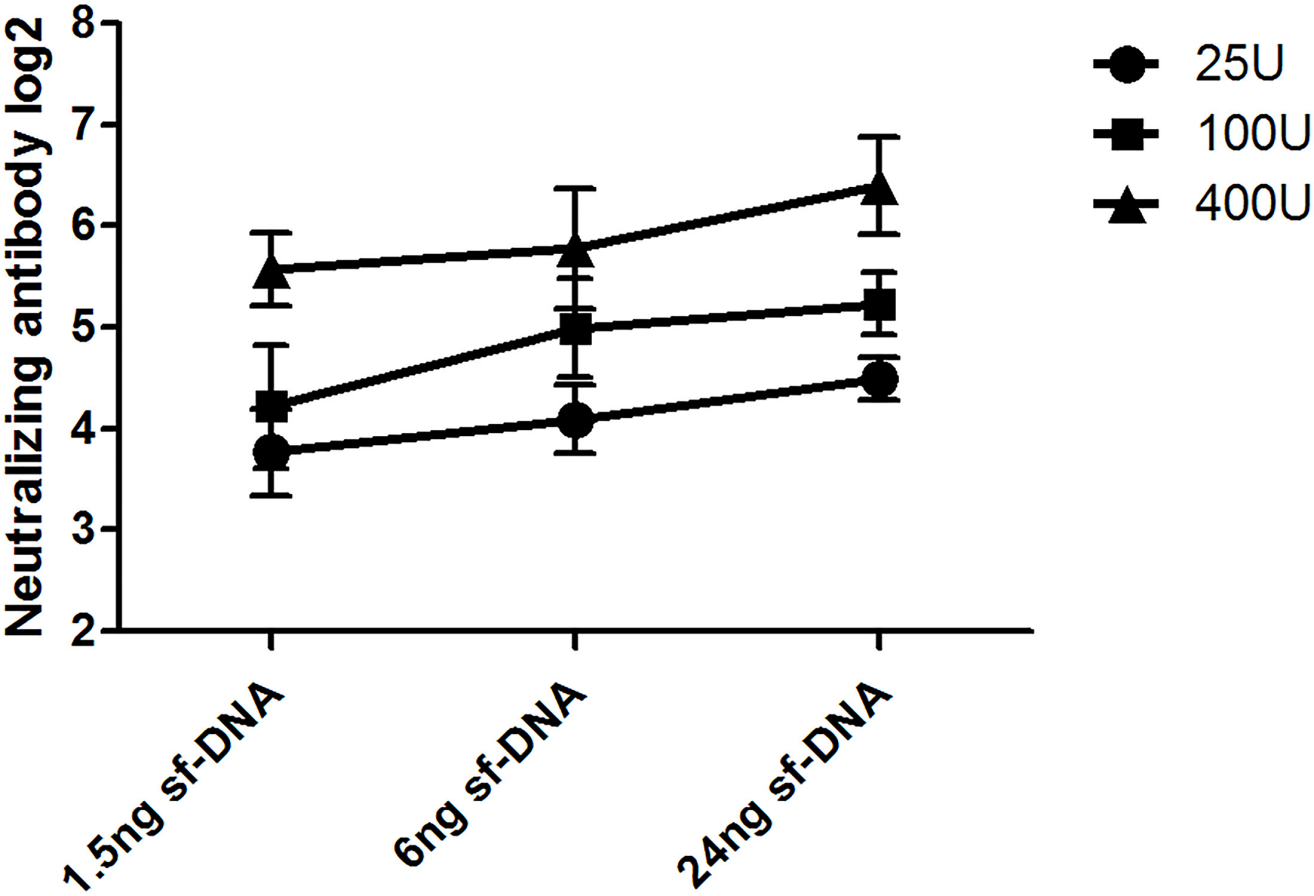
NTAb titer after vaccination with EV71 inactivated liquid bulk adjuvanted with sf-DNA. Six to eight week-old femal C57BL/6 mice (n = 10 per group) were injected with 25U, 100U and 400U EV71 inactivated liquid bulk adjuvanted with three concentrations of sf-DNA (1.5ng, 6ng, 24ng), respectively. The NTAb level was tested 7d after vaccination, and NTAb titers increased with the rising of sf-DNA concentration in all three dosages.

Since TLR9 gene knockout mice showed reduced production of NTAb after vaccination with EV71 inactivated liquid bulk, a question naturally emerged in our mind whether sf-DNA could be used to improve the effect of new EV71 inactivated vaccine. Three concentrations of EV71 inactivated liquid bulk (25U, 100U, 400U) were adjuvanted with three concentrations of sf-DNA (1.5ng, 6ng, 24ng), respectively. As showed in Fig 10, the NTAb titers increased with the rising of sf-DNA concentration in all three dosages.

## Discussion

In this paper we have shown TLR4 and TLR9 were activated in response to the vero-derived EV71 inactivated liquid bulk. TLR4 was considered to recognize the structural protein of virus, such as the fusion protein of respiratory syncytial virus [30] and the envelope protein of mouse mammary tumor virus[31]. Thus, it is not surprising that TLR4 is involved in the immune response to EV71 inactivated Ag, due to the fact that main components of this inactivated Ag is the structural protein of EV71 virus. A recent study also showed that EV71 VLP induced the activation and maturation of mDC through TLR4 signaling[26], which was consistent with the results of this study. Interestingly, TLR9 has been found to play an important role after vaccination with the EV71 inactivated Ag as well as TLR4. TLR9 was reported to recognize DNA that contained sequences rich in CpG-DNA motifs like bacterial genomes[32] and viral DNA[33]. EV71 belongs to the *Picornaviridae* family using RNA as its genetic material[34], thus gDNA from host cell is regarded as the only source of DNA components in the EV71 vaccine.

Vero cell has been recommended as one cell substrate for production of human vaccines, however, a specification of the residual vero cell DNA is strictly enforced in consideration of the safety of biological products[22, 23]. WHO recommends that the final DNA content in per dose of the vaccine products should be less than 10ng[35]. The US FDA mandates that residual DNA content of each dose of biological products should be no more than 100pg. The Chinese Pharmacopoeia requires that residual exogenous DNA in vero cell culture-derived vaccines should be no more than 100pg per dose. IPV vaccine manufactured by Pasteur Co., Ltd, using vero cells requires residual DNA content to be under 10pg per dose[36]. Nuclease treatment is proved to be a convenient, economic and efficient method to remove host DNA[19, 20], and has been approved to be used in several vaccines, such as HAV vaccine (VAQTA^®^, MSD, USA)[37], H1N1 vaccine (Celvapan^®^, BAX, USA)[38], as well as the new EV71 inactivated vaccine (Inlive^®^, Sinovac, China). It has been reported that residual host cell DNA decreased to an extremely low levels (<100pg/ml) after treated with Benzonase in the final product of vero-cell culture-derived human rabies vaccine[20].

Short-fragment DNA is a theoretical by-product after nuclease digestion. But it is too short to be detected by methods like dot blot hybridization assay and q-PCR recommended in the Chinese Pharmacopoeia. In this study, we chose Qubit dsDNA HS assay to detect the sf-DNA in the EV71 vaccine, due to its high sensitivity, suitable detection range on DNA length[39–41] and most importantly, no other method available at present. Purified 20~50bp sf-DNA was read 2500pg/ul by the Qubit, which proved that the method could be used to detect sf-DNA.

Results in this study demonstrated the upregulation of TLR9 was triggered by sf-DNA, even though the changes of the cytokine levels after sf-DNA stimulation were not tested due to it was difficult to speculate the specific pathway activation from cytokine expression profiles. Hsiao has also reported the released endogenous DNA from dead cells mediated protection of EV71 infection in mice through TLR9 signaling[27]. Interestingly, our data showed EV71 inactivated vaccine adjuvanted with sf-DNA can promote the generation of EV71 neutralization antibodies. This means that sf-DNA has a potential adjuvant effect. Due to the benzonase cut DNA relatively nonspecific, the sf-DNA mentioned here is a mixture of numerous DNA fragments with different sequences. Thus the sequences of the bioactive sf-DNA should be identified in future study.

In summary, we have stated a fact that residual sf-DNA in the EV71 vaccine could induce the upregulation of TLR9 and then promote the generation of EV71 NTAb. However, there is a limitation in this study. The functions of other TLRs, particularly TLR3 and TLR7, in response to the EV71 vaccine still need be evaluated in further study.

## Abbreviations

sf-DNA: short-fragment DNA
TLR: toll like receptor
EV71: enterovirus 71
NTAb: neutralizing antibodies
CPE: cytopathic effect
DC: dendritic cells
gDNA: genomic DNA
